# Quasi-periodic concave microlens array for liquid refractive index sensing fabricated by femtosecond laser assisted with chemical etching

**DOI:** 10.1038/s41598-018-20807-1

**Published:** 2018-02-05

**Authors:** F. Zhang, C. Wang, K. Yin, X. R. Dong, Y. X. Song, Y. X. Tian, J. A. Duan

**Affiliations:** State Key Laboratory of High Performance Complex Manufacturing, School of Mechanical and Electrical Engineering, Central South University, Changsha, 410083 China

## Abstract

In this study, a high-efficiency single-pulsed femtosecond laser assisted with chemical wet etching method has been proposed to obtain large-area concave microlens array (MLA). The quasi-periodic MLA consisting of about two million microlenses with tunable diameter and sag height by adjusting laser scanning speed and etching time is uniformly manufactured on fused silica and sapphire within 30 minutes. Moreover, the fabricated MLA behaves excellent optical focusing and imaging performance, which could be used to sense the change of the liquid refractive index (RI). In addition, it is demonstrated that small period and high RI of MLA could acquire high sensitivity and broad dynamic measurement range, respectively. Furthermore, the theoretical diffraction efficiency is calculated by the finite domain time difference (FDTD) method, which is in good agreement with the experimental results.

## Introduction

Monitoring refractive index (RI) of liquid is critical in the field of optofluidics, medical, chemical, industrial and environmental applications^1,2^. In recent years, a lot of optical sensors for detecting liquid RI have been proposed by the advantages of low cost, wide dynamic range, high accuracy, high reliability and label-free^3–5^. According to their operation principle, the optical sensors can be divided into two broad categories: goniometric and interferometric. The former contains Abbe refractometer^6^ and Wollaston cube prism refractometer^7^, while the later includes Mach-Zehnder interferometer^8^, Fabry-Perot interferometer^9^, young double slit-based refractometer^10^ and micro-ring resonator^11^. However, most of those sensing approaches require sophisticated facilities including beam splitter, prism and spectrometers and so on. Hence, a variant of optical sensors that have been drawn significant attentions are based on the use of diffraction optical element (DOE)^12–14^. Kumawat *et al*. proposed a refractive sensor based on the interferometric mixing of multiply reflected diffraction orders using transparent phase gratings^15^. Zhou *et al*. reported a set of bioactive DOE microfabricated using functionalized silk fibroin films for hydration sensing, biological concealment and therapeutic treatment^16^. Xu *et al*. presented an optofluidic sensor for monitoring the change of the liquid RI with high sensitivity based on a 2D grating integrated inside a hemispherical fluid chamber^17^. Walia *et al*. demonstrated a cost-effective RI sensor by measuring color changes from 2D silicon nanowires arrays using a trichromatic RGB decomposition^18^. In addition, being as one of most important DOE, microlens array (MLA) has also been used for RI sensing with high sensitivity^19,20^.

Recently, many methods for the fabrication microlens array have been proposed, such as reactive ion etching^21^, thermal reflow^22^, self-assembly method^23^, fictive temperature modification^24^, mask replication^25^, and so on. However, the manufacturing processes of MLA mention above are very complex and requiring precise mask. Besides, it is intricate to get the desired profiles of the microlenses in several micrometers. Lately, femtosecond lasers have proved to be a promising tool for controllable, flexible, mask-less and nanometer accuracy processing^26,27^. Due to the short laser pulse time as compared with most characteristic times of chemical and physical processes and the extremely high irradiance^28,29^, femtosecond laser pulses are promising for unique processing of microlens array. Unfortunately, the efficiency for large-area MLA fabrication by femtosecond laser point-by-point scanning is slightly low^30^. Therefore, it is still a challenge to develop a high-efficient, low-cost, and tunable strategy for fabricating large-area uniform MLA applying in liquid RI sensing.

In this paper, a high speed scanning approach to fabricate large-area high quality concave MLA by chemical-etching-assisted femtosecond laser irradiation is proposed. The MLAs on the fused silica and sapphire are formed under single pulse laser irradiation after being treated by hydrofluoric (HF) acid solution and potassium hydroxide (KOH), respectively. The scanning electron microscope (SEM) and laser confocal microscopy (LCM) have been used to characterize the surface microstructures of MLA. In order to obtain bright focusing and clear imaging of MLA, the diameter, sag height, focal length, numerical aperture and period could be properly controlled by laser scanning speed and etching time. In addition, the uniform MLA has been employed to sense liquid RI based on optical diffraction, which is confirmed by the finite difference time domain (FDTD) method.

## Results

**Figure 1:**
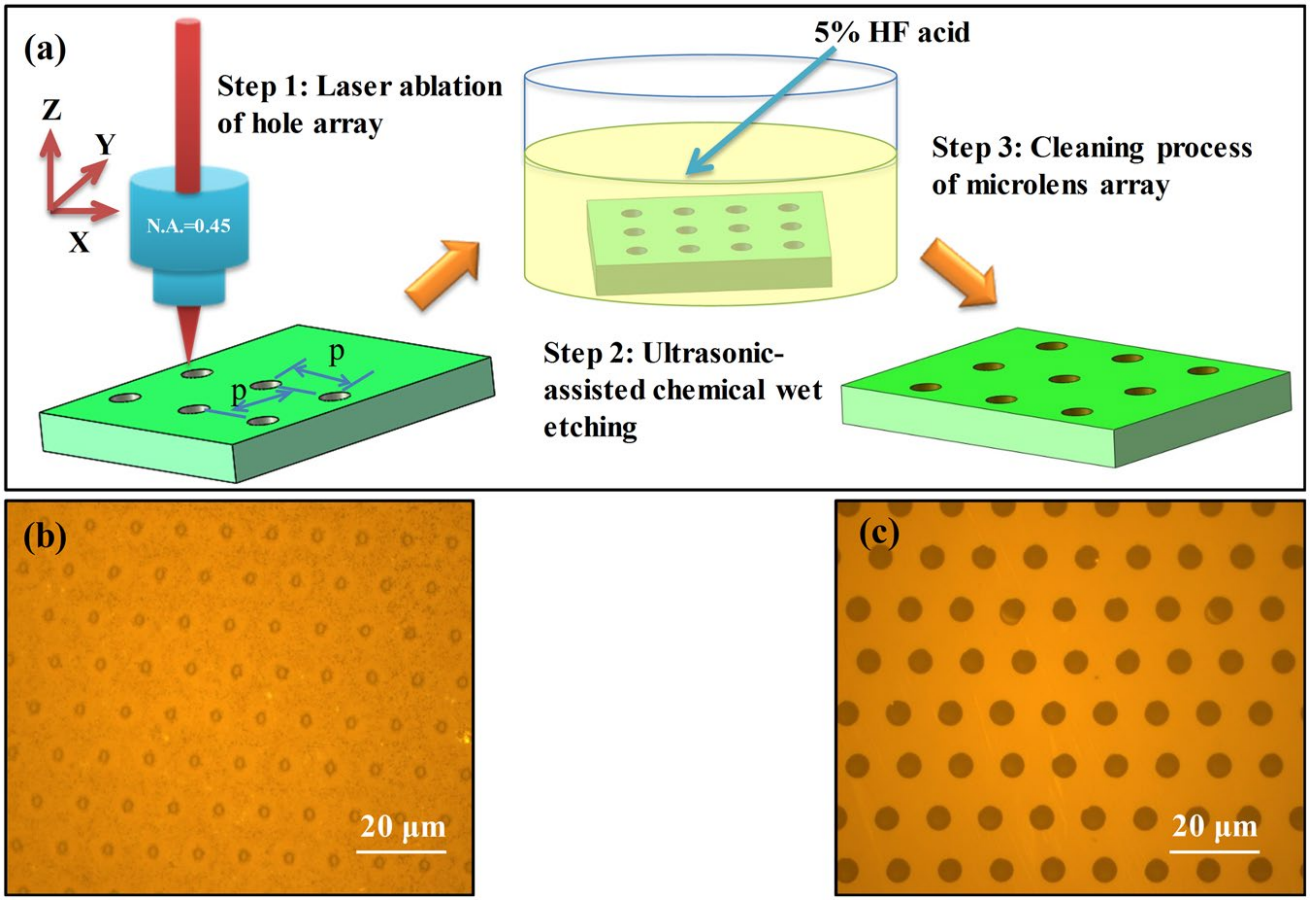
(**a**) Schematic diagram of fabrication process for fused silica MLA, where p is defined as the period of MLA. The morphology of the sample surface captured by LCM: (**b**) before etching; (**c**) after etching.

**Figure 2:**
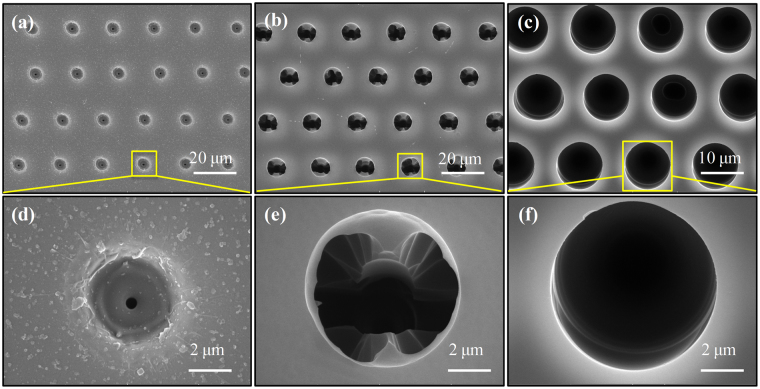
SEM image of fabricated fused silica MLA etching at different time**:** (**a**) 0 min; (**b**) 10 min; and (**c**) 30 min. The pulse energy is 5 μJ.

Figure 1(b) and (c) illustrate the surface morphology of fused silica before etching and after 30 min etching, respectively. It is observed that the hole array with crack and incomplete circular aperture goes against the focusing and imaging before chemical etching. However, the concave microstructures with perfect spherical morphology after etching can be treated as microlens array (MLA) for high quality imaging. Therefore, being as one of the key parameters for the microlens formation, the etching time has been carefully investigated. Figure 2 exhibits SEM image of the MLA formation process at different chemical etching time. By reason of the fast scanning speed, the ablation craters separate from each other, leading a single pulse to form a microlens. The nanostructures in the laser ablation area such as the grooves and protrusions observed in Fig. 2(d) are generated by the nonlinear process during femtosecond laser-material interactions^31^. These structures and defects will enlarge the interface of the sample’s surface and etching solutions, resulting in accelerating the etching process in the hole. The intrinsic atomic-scale structures of fused silica can be described as a broad random network consisting of repeated Si–O bonds. Silica is known to be attacked by aqueous HF through the following reaction scheme^32^:

1 $$\equiv {\rm{Si}}-{\rm{{\rm O}}}-{\rm{Si}}\equiv \,\,+\,{{\rm{{\rm H}}}}^{+}{{\rm{F}}}^{-}\,\,\to \,{\rm{Si}}-{\rm{OH}}\,\,+\,\,\equiv {\rm{SiF}}$$

Due to the deformed configuration of their valence electrons, the decrease in bridging bond angle in densified silica increases the reactivity of the oxygen atoms. The increase in reactivity is mainly responsible for a greater susceptibility to the HF etching in the modified zones with respect to the unmodified zones. When the etching time is 10 minutes, the hole diameter and depth in Fig. 2(b) are apparently increasing comparing to those without etching. Nevertheless, incomplete etching emerges at the bottom of the hole in Fig. 2(e), which indicates that 10 minutes etching time is not enough to form high quality MLA. After 30 minutes chemical etching, the diameter of hole further enlarges, as shown in Fig. 2(f). Consequently, the high quality and good uniformity concave quasi-period MLA has been formed on the substrate surface in Fig. 2(c). This indicates that the high speed scanning method is able to manufacture smooth and uniform microstructures. Nevertheless, due to the insufficient accuracy and the vibration of the high speed moving 3D translation, the microlens elements are quasi-regularly arranged^33^.

The detailed data of the diameter, sag height (depth in the cross-section profile), focal length, numerical aperture (NA) and average surface roughness (Ra) during etching process for different period MLA fabricated by 25 μJ laser pulse are listed in Table 1. The fused silica microlens diameter and sag height are measured from ten microlenses, while the focal length and numerical aperture are obtained from theoretical equation. The surface roughness average is acquired from laser confocal microscopy (LCM) measurement within 250 × 250 μm^2^ scanning square area. Firstly, the diameter and sag height are apparently enlarged with the increase of etching time for any scanning speed. Meanwhile, the enhancement of scanning speed will slightly lead to the enlargement of diameter and reduction of sage height. Secondly, the focal length (*f*) and NA of MLA can be deduced from diameter (*D*) and sag height (*h*) following the equations^34^,

2 $$f=\frac{{h}^{2}+{(D/2)}^{2}}{2h(n-1)}$$

3 $$NA=\frac{D}{2f}$$

where *n* ( = 1.45) is the value of the refractive index of the fused silica at wavelength of 632 nm. Hereby, the focal length f slightly declines as the etching time rises, and obviously goes up by accelerating the scanning speed, which is due to the increase of the MLA’s diameter. The value ranges of focal length and NA of fabricated MLA are 12.05–33.93 μm and 0.11–0.33, respectively. Such low values of focal length and NA are attributed to the reason that the small diameter of microlens possessing small curve radius can only gather small amount of light. The MLA displays good surface smoothness, and the average surface roughness (Ra) is less than 0.4 μm within 250 × 250 μm^2^ scanning square area. For any laser scanning speed, the value of Ra firstly decreases then increases as the etching time expands. The chemical etching has the effect of smoothing surface on the laser ablated rough area^26^. As the etching time goes up, the diameter and sag height will been apparently enhanced, which result in the enlargement of Ra as the surface fluctuates.

**Table 1:** The detailed diameter, sag height, focal length, numerical aperture (NA) and average surface roughness (Ra) during etching process for MLA with different period fabricated by 25 μJ laser pulse.

Scanning speed (mm/s)	Etch time (minutes)	Diameter (μm)	Sag height (μm)	Focal length (μm)	NA	Ra (μm)
10	0	6.41	1.02	12.32	0.26	0.301
	10	6.92	1.22	12.25	0.28	0.188
	20	7.38	1.43	12.16	0.30	0.363
	30	8.09	1.81	12.05	0.33	0.398
20	0	6.90	0.73	18.92	0.18	0.201
	10	7.95	1.01	18.50	0.21	0.119
	20	8.20	1.34	15.42	0.26	0.145
	30	8.70	1.61	14.84	0.29	0.292
30	0	8.05	0.54	33.93	0.11	0.105
	10	8.60	0.87	24.58	0.17	0.100
	20	9.94	1.18	24.56	0.20	0.160
	30	10.8	1.41	24.54	0.22	0.197

## Discussion

**Figure 3:**
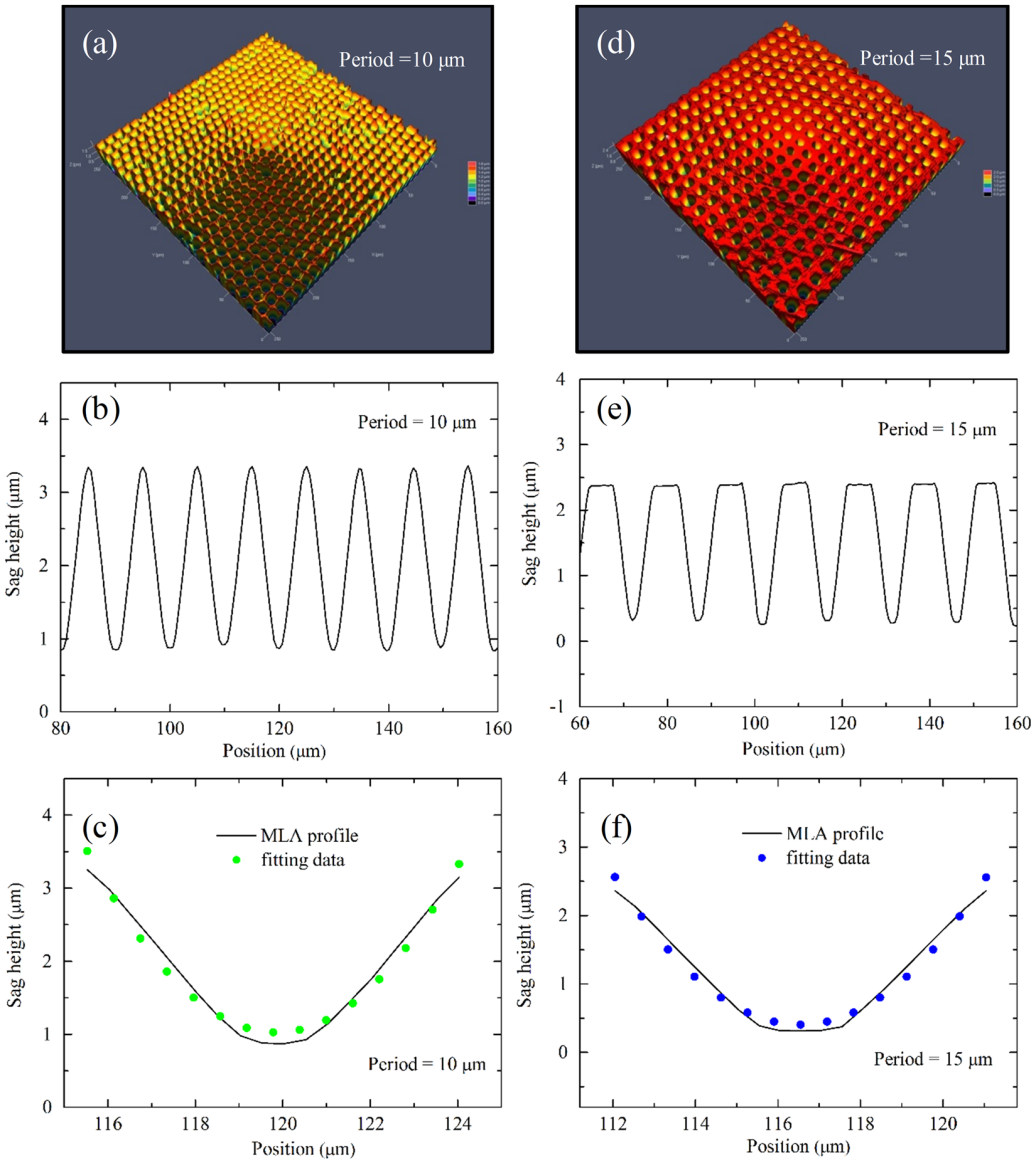
The 3D morphology, cross section profiles and the enlarged view of the profiles for the concave fused silica MLA after chemical etching with a period of (**a–c**) 10 μm and (**d–f**) 15 μm, respectively.

Figure 3 exhibits the LCM 3D surface morphologies and cross-sectional profiles of the MLA on fused silica with a period of 10 μm and 15 μm fabricated by 5 μJ pulse laser energy, respectively. Figure 3(a) and (d) demonstrate the perfect surface uniformity of MLA with microlenses regularly spreading out on the surface of samples in the fixed spacing. Figure 3(b) and (e) show the cross-section profiles of several microlenses. In consequence, the average aperture diameters and sag heights of single microlens are 8.5 μm and 2.5 μm for the period of 10 μm, while 8.9 μm and 2 μm for the case of 15 μm, respectively. Moreover, according to the experiments and theoretical fitting section in Fig. 3(c) and (f), the surface of concave microlens can be regarded as parabolic, since the root-mean-square error between the measurement and fitting data is less than 0.025 μm. The parabolic shape of a microlens is suitable for minimizing spherical aberration in optical device^35^.

**Figure 4:**
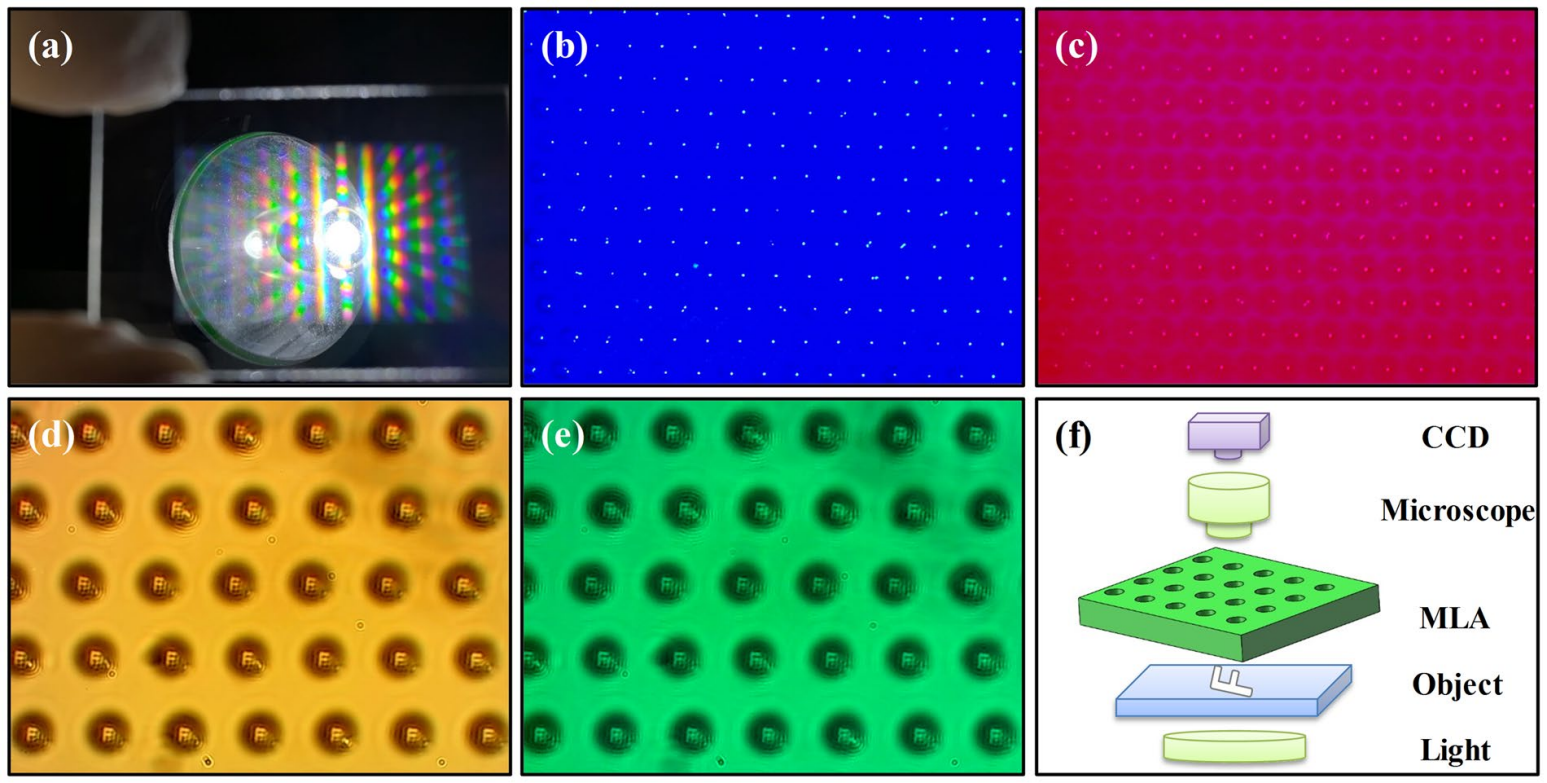
(**a**) The digital photo of the fabricated MLA (p = 15 μm) sample illuminated by white light source; Focal image of MLA sample illuminated by the (**b**) 441 nm and (**c**) 632 nm spot light source; Imaging properties of the MLA sample under illumination with (**d**) halogen light source and (**e**) 532 nm light source; (**f**) Schematic of the optical system for the measurement of the “F” imaging of the MLA sample.

A large-area transparent MLA covering 2 × 1 cm^2^ area consisting of about 2 million microlenses has been fabricated on the fused silica sheet in Fig. 4(a). The total manufacture procedure only lasts about 30 minutes, which states that high speed scanning mode provides an efficient approach to fabricate large-area MLA. Once the white light illumination in Fig. 4(a), multiple rainbow patterns in a quasi-period arrangement are observed surrounding the non-diffracted light (zero-order). The blue, green, yellow and red light on the rainbow pattern are successively diffracted from shorter to longer distances due to the refractive index for red light in fused silica is greater than that for blue light. The similar diffracted rainbow patterns from a hexagonal MLA illuminated by the broadband light have been previously reported^36^. Figure 4(b) and (c) display the quite uniform array of bright spots on the false focal plane of the MLA under illumination of blue light source (441 nm) and red light source (632 nm), which illustrates that the MLA performs good consistency of the focal lengths. An opaque mask with a transparent letter “F” has been inserted between the MLA and a bright light source, as exhibited in Fig. 4(f). And the false images from MLA through objective lens are captured by a CCD camera, as demonstrated in Fig. 4(d) and (e) for different light source. The clear images of “F” in size and shape are homogeneous in Fig. 4(d) for halogen light source, expressing the uniformity of the microstructures and the superior imaging feature of the MLA. For monochromatic light of green (532 nm) in Fig. 4(e), the false image of “F” is also efficient. The fine image property and uniform focal length facilitates the MLA eligible to be served as multi-functional DOE devices.

**Figure 5:**
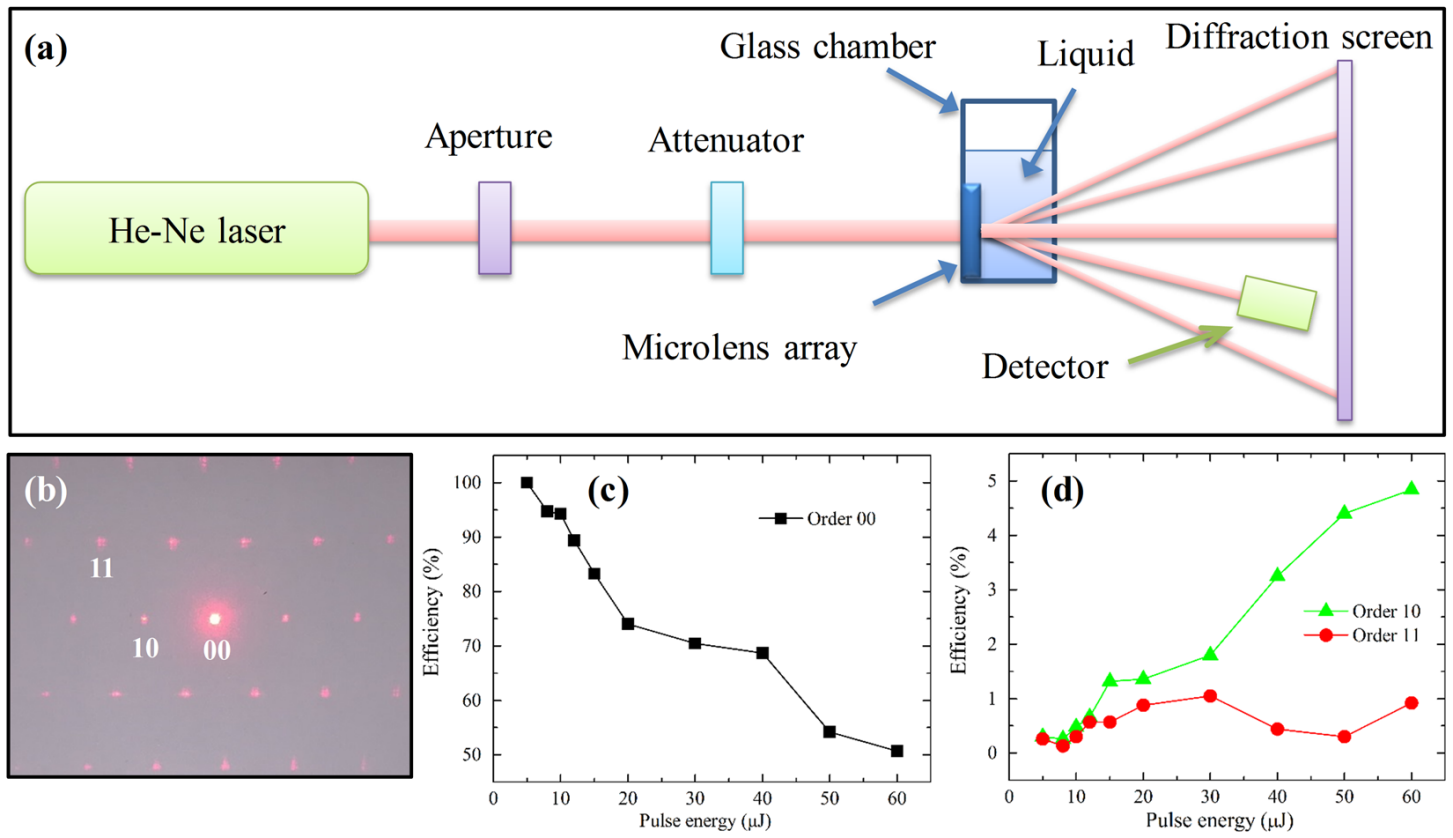
(**a**) Schematic of the diffraction sensing setup; (**b**) Photograph of exemplary diffraction pattern on diffraction screen; The diffraction efficiency of (**c**) order 00, (**d**) order 10 and order 11 for fused silica MLA without liquid as a function of laser pulse energy.

Figure 5(a) is a schematic of the experiment setup to monitor the liquid RI surrounding the MLA in a glass chamber. The beam from a He-Ne laser (632 nm) with a power of 2 mW is diffracted by the MLA to project a diffraction pattern on the screen, as presented in Fig. 5(b). The direct transmission in diffraction pattern is referred as order 00. And the nearest and next-nearest neighbor of order 00 is referred as order 10 and order 11, respectively. As is known, the laser pulse energy is a crucial parameter in MLA based liquid RI sensing to control the diffraction efficiency. Hence, Fig. 5(c) and (d) depict the change of different order diffraction efficiency of MLA on fused silica as a function of laser pulse energy at a fixed period of 15 μm assisted with 30 minutes chemical etching. As the laser pulse energy raised from 5 μJ to 60 μJ, the diffraction efficiency of order 00 monotonously decreases due to the enhancement of surface scattering and absorption^37^. Otherwise, the diffraction efficiency of order 10 distinctly raises with the increase of laser pulse energy, which could be devoted to the enlargement of optical phase shift on account of augment of microlens sag height under high power femtosecond laser irradiation^38^. The efficiency of order 11 appears oscillation at a low value (less than 1%) versus to laser pulse energy.

**Figure 6:**
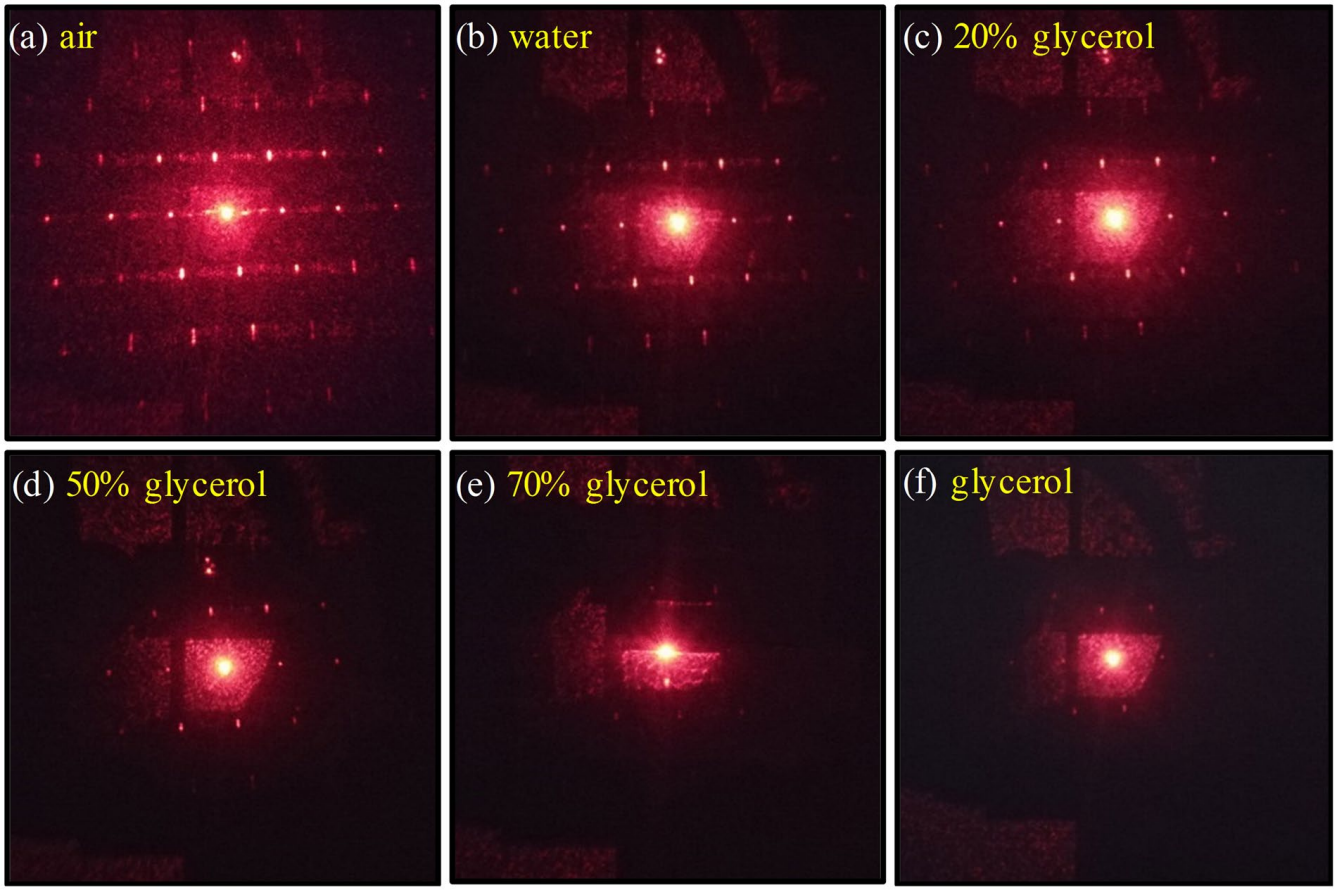
The diffraction pattern of fused silica MLA measured in different conditions: (**a**) air, (**b**) water and glycerol solution with concentration of (**c**) 20%, (**d**) 50%, (**e**) 70% and (**f**) 100% by volume.

To verify the RI detection of fabricated setup, the glycerol solutions at different concentrations have been prepared as liquids with different RI. Also, the relationship between the concentration and RI of glycerol solutions could be referred to ref.^39^. Figure 6 shows the diffraction patterns of the MLA on fused silica when air, water and glycerol solutions with different concentrations are injected into the glass chamber. As the concentration is enhanced, the diffraction pattern especially in high-order apparently gets weaker corresponding to liquid RI from 1 to 1.47, which can be elaborated by the fact that the liquid RI is increasingly close to fused silica RI (n = 1.45). Accordingly, the liquid RI in glass chamber can be detected by the relative change of the MLA’s diffraction pattern. In addition, Xu *et al*. have proved that although the diffraction angle could be used to monitor the RI, the diffraction power or diffraction efficiency is more sensitive for liquid RI sensing^40^. Hence, the relationship between the diffraction efficiency and the change of liquid RI is carefully investigated in this study.

**Figure 7:**
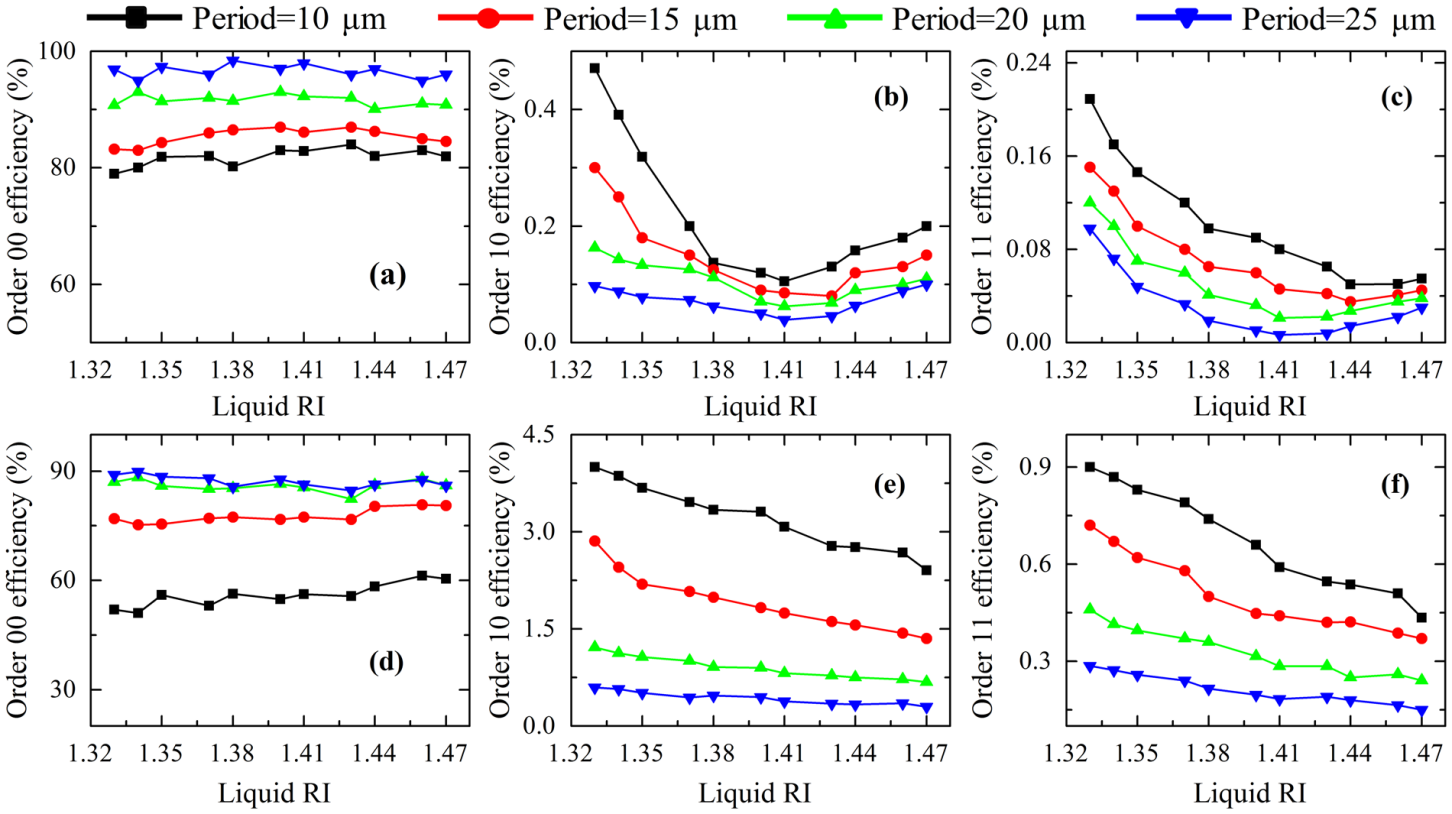
The diffraction efficiency of order 00, order 10 and order 11 as a function of liquid RI for (**a–c**) fused silica MLA and (**d–f**) sapphire MLA with different period.

In order to quantitatively describe the liquid RI sensing, the diffraction efficiencies of the MLA on fused silica (RI = 1.45) and sapphire (RI = 1.77) with different period have been measured as a function of liquid RI ranging from 1.33 to 1.47, as plotted in Fig. 7. The upper panel of Fig. 7 shows the diffraction efficiency of order 00, order 10 and order 11 for fused silica, while it is the case of sapphire in the lower panel. It is found that the order 00 efficiency of fused silica and sapphire obviously increases as the period raises, but it is little affected by liquid RI. It is attributed to that the smaller period MLA with rougher surface (referred to Table 1) could diffuse more light intensity, which results in few diffraction power received by the detector. As for the order 10 and order 11 efficiency of fused silica (RI = 1.45), it decreases first and then increases as liquid RI increases. When the liquid RI is about 1.45, it reaches the minimum. While for sapphire (RI = 1.77), the order 10 and order 11 efficiency almost linearly and monotonically decreases with the increase of liquid RI. It could be elaborated that the diffraction effect or diffraction intensity will decline when the liquid RI is close to the MLA RI. Hereby, the slope percent in the liquid RI ranging from 1.33 to 1.4 can be introduced to represent the sensitivity. Obviously, the high sensitivity or steep slope appears in the cases with small period. Furthermore, the sensitivity of 10.63/RIU for order 10 of fused silica with a period of 10 μm is higher than that of 2.5/RIU for sapphire in spite of its better linearity and large value of efficiency. Nevertheless, the sapphire MLA with higher RI (1.77) can broaden the measurement range of liquid RI from previous 1.45 to current 1.77.

**Figure 8:**
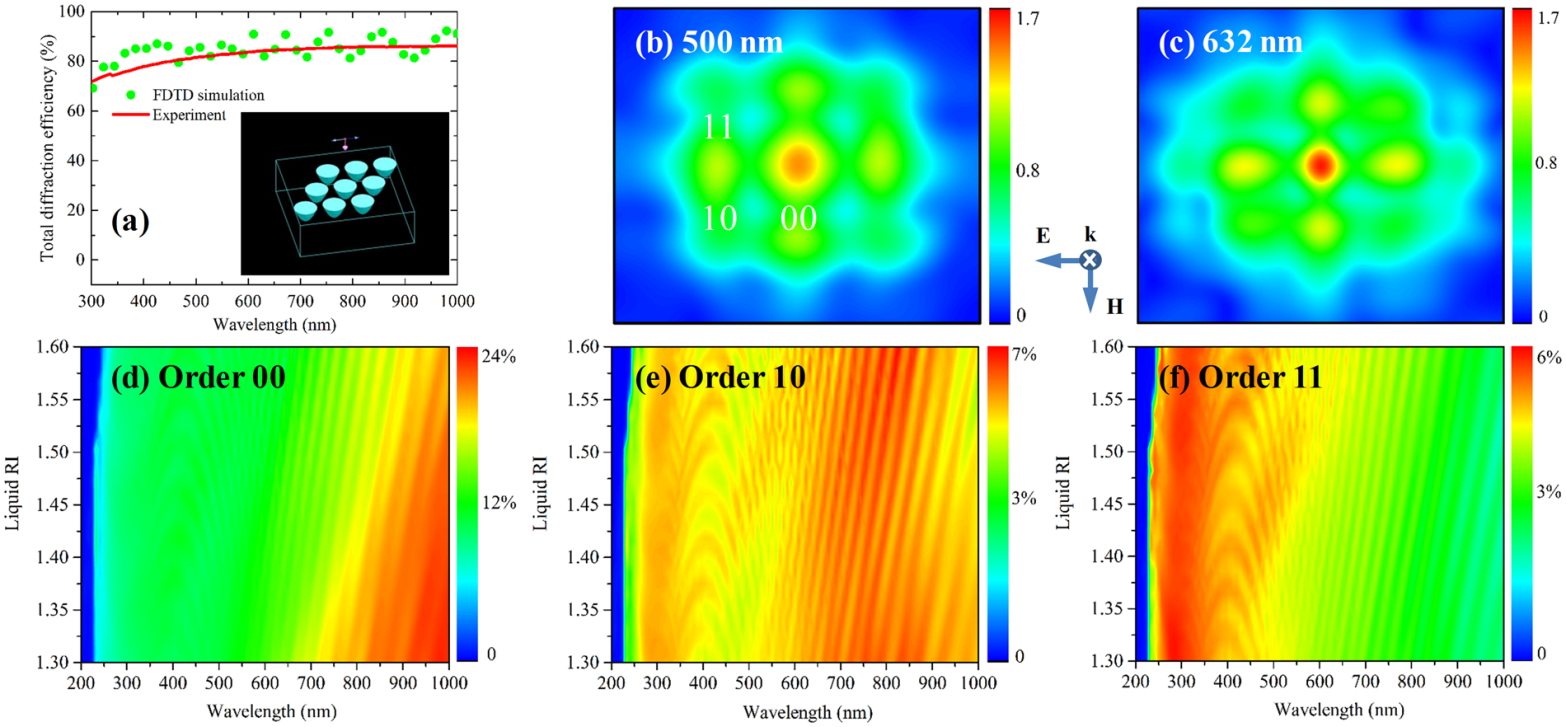
(**a**) The experimental and simulated total diffraction efficiency spectra of fused silica MLA. The inset is the 3D MLA model in FDTD simulation; Calculated diffraction pattern when environment RI is 1 illuminated by (**b**) 500 nm and (**c**) 632 nm light source; Contour plots of the calculated (**d**) order 00, (**e**) order 10 and (**f**) order 11 diffraction efficiency spectra of fused silica MLA for different liquid RI.

Figure 8(a) displays the measured and calculated total diffraction spectra for the fused silica (RI = 1.45) MLA with a period of 10 μm in air (RI = 1). The 3D simulation model of the MLA used in this calculation is also shown in the inset of Fig. 8(a). Generally, the calculated diffraction efficiency approximately reveals a similar trend to the measured results despite the presence of a discrepancy at some wavelengths between the calculated and measured curve. This is due to incompletely matching the geometric simulation model to the actual fabricated samples. The total diffraction efficiency is decreased as the wavelength varies from 1000 nm to 300 nm, which is devoted to the intrinsic absorption of fused silica in ultraviolent region. Figure 8(b) and (c) exhibit the simulated diffraction pattern (electric field intensity *E*_*x*_ distribution) of the MLA in air (RI = 1) illuminated by 500 nm and 632 nm light, respectively. The diffraction patterns in nearly symmetrical distribution emerges diffraction order 00, order 10 and order 11. The diffraction intensity for the 632 nm light is larger than that for the 500 nm case, which is in agreement with the spectra in Fig. 8(a). Generally, it declares that the proposed simulation model and acquired diffraction efficiency are reasonable and reliable. Therefore, the simulated diffraction efficiency spectra of the fused silica MLA for order 00, order 10 and order 11 could be obtained in liquid with different RI, as shown in Fig. 8(d–f), respectively. Clearly, the large diffraction efficiency region in Fig. 8(d) is shifted towards the longer wavelengths with enlarging the liquid RI on account of the diffraction losses caused by the higher order waves^41^. The diffraction efficiency of order 10 at wavelength of 632 nm is slightly decreased with the increase of the liquid RI, which is in accordance with Fig. 7(b). Consequently, the MLA structures on the surface of fused silica could significantly sense the change of liquid RI over a broad wavelength region. The sensor is versatile since various measuring ranges and numerous levels of sensitivities could be covered by selecting different MLA or illuminating wavelength^42^.

## Conclusions

In summary, we have developed a high-efficiency method to fabricate large area concave MLA by femtosecond laser assisted with chemical wet etching. The quasi-periodic MLA with about two million microlenses is uniformly formed on fused silica or sapphire in a short time. In addition, the diameter, sag height, focal length, numerical aperture and period could be facilely regulated through laser scanning speed and etching time. The bright focusing and clear imaging properties of the MLA availably demonstrate the excellence optical performance. Thereafter, the MLA has been employed to sense the liquid RI based on optical diffraction. To get high sensitivity and broad dynamic measurement range, the small period and high RI of MLA could be used, which indicates that the proposed approach for measuring the liquid RI is feasible. Moreover, its sensing mechanism has been investigated by FDTD method. The theoretical optical calculation results have revealed a similar trend to the experimentally measured data. It is anticipated that the liquid RI sensor based on MLA will broaden potential applications of diffraction optical element in microscale optical devices and miniaturized sensors.

## Methods

The fused silica and sapphire (c-plane) sheets (2 cm × 3 cm × 0.1 cm) with double-side polishing are mounted on a 3-axis linear translation stage (Newport, Inc.), which is controlled by a computer program and has a precision of 20 nm in the x, y and z directions. The regenerative amplified Ti:sapphire laser system (Spectra Physics Spitfire) can generate 120 fs Gaussian laser pulses with a central wavelength of 800 nm and repetition rate of 1 kHz. The laser pulse energy is attenuated through a neutral density attenuator before an objective lens (20× , NA = 0.45, Olympus). The samples are translated in x direction vertical to the laser beam at a speed of *p* mm/s (*p* varying from 10 to 25). As the laser system is a pulsed laser with repetition of 1 kHz, the average separation of the concave hole is *p* μm. In order to form hole array in Fig. 1(b), a square is fabricated along the y direction with a *p* μm transverse distance between scanning lines, as shown in Fig. 1(a). Then, a quasi-periodic array of laser induced holes is generated on the surface of sample, the period (*p*) of which can be controlled by computer programming. Subsequently, the fused silica and sapphire are immersed in 5% hydrofluoric (HF) acid solution and 40% potassium hydroxide (KOH) at room temperature, respectively^43^. In order to acquire uniform microstructures, an ultrasonic bath depicted in Fig. 1(a) is adopted to wipe out the bubbles generated at liquid-solid interface during chemical wet etching process. When the smooth concave surfaces are formed in Fig. 1(c), the samples are rinsed in de-ionized water and cleaned in an ultrasonic bath.

The surface microstructures are observed by a scanning electron microscope (SEM, Tescan, MIRA 3 LMU). The 3D surface morphology is measured by an Axio LSM700 laser confocal microscopy (LCM, Zeiss). The transmittance efficiency is obtained by measuring the light power before and after the vertically pass through sample. He-Ne laser (632 nm) is employed as light source. A spectrophotometer is employed to acquire transmission spectra in the wavelength of 300–1000 nm.

For the theoretical optical analysis of the MLA, the simulation is performed based on finite difference time domain (FDTD) method by using a commercial software (Lumerical FDTD Solutions 8.6). In the simulations, the mesh size is set as 200 point for wavelength range 200–1000 nm when calculating the transmission. Further, when calculating the *E* field distribution, the mesh size is set as 15 nm in order to obtain high resolution plot. The perfectly matched layer (PML) boundary conditions are adopted.

## Electronic supplementary material

Supplementary Information
