# Supplementary material for: Quasi-periodic concave microlens array for liquid refractive index sensing fabricated by femtosecond laser assisted with chemical etching
Source: Sci Rep. 2018 Feb 5;8:2419. doi: 10.1038/s41598-018-20807-1 (PMC5799298; doi:10.1038/s41598-018-20807-1)
Supplement: Supplementary file 1 — Supplementary Information [file 41598_2018_20807_MOESM1_ESM.doc]

**Quasi-periodic concave microlens array for liquid refractive index sensing fabricated by femtosecond laser assisted with chemical etching**

**Fan Zhang, Cong Wang*, Kai Yin, Xinran Dong, Yuxin Song, Yaxiang Tian and Ji'an Duan***

State Key Laboratory of High Performance Complex Manufacturing, School of Mechanical and Electrical Engineering, Central South University, Changsha 410083, China

Correspondence should be addressed to C.W. (email:wangcong@csu.edu.cn) or J.A.D. (email:duanjian@csu.edu.cn)

**Supplementary Materials**

The laser pulse energy dependence of the diameter and height of the microlens have also been investigated. According to Figure S1, the diameter and height of the microlens are both increased with the increase of laser energy. Therefore, the lens profile could be controlled by adjusting laser energy. However, the surface roughness is obviously increased in the cases with high laser pulse energy [S1]. In addition, the spatter around the microlens induced by large laser energy is not beneficial to the optical performance of MLA [S2].


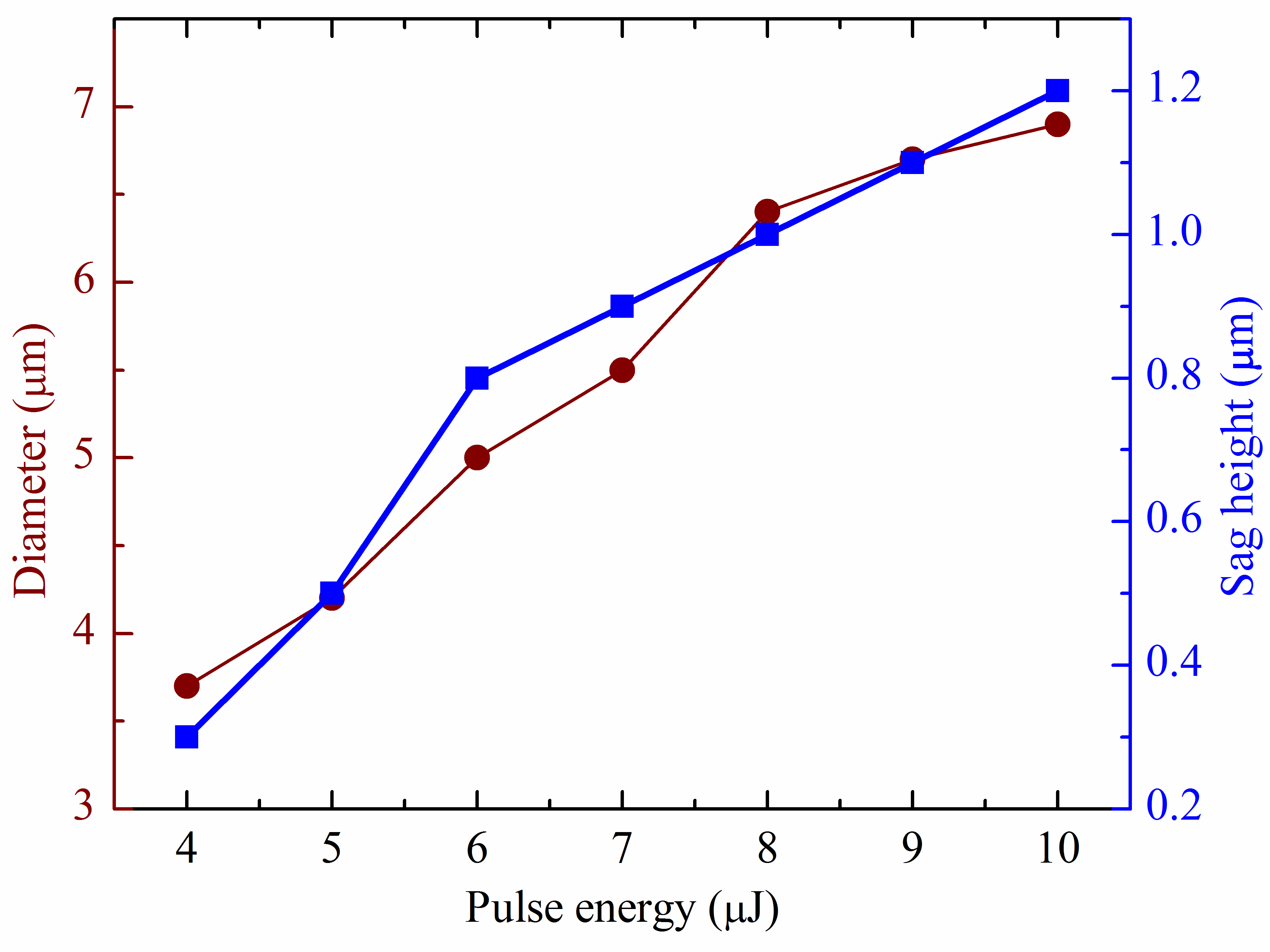


Figure S1. Pulse energy dependence of the diameter (circle markers) and sag height (square markers) fabricated by femtosecond laser.

The initial beam profile is a Gaussian type energy distribution, as shown in Figure S2. During laser material interactions, the beam profile would be changed, which could be described by the plasma model with the consideration of laser particle-wave duality [S3]. The 3D and side view of the beam profile during laser material interactions are displayed in Figure S3, respectively.


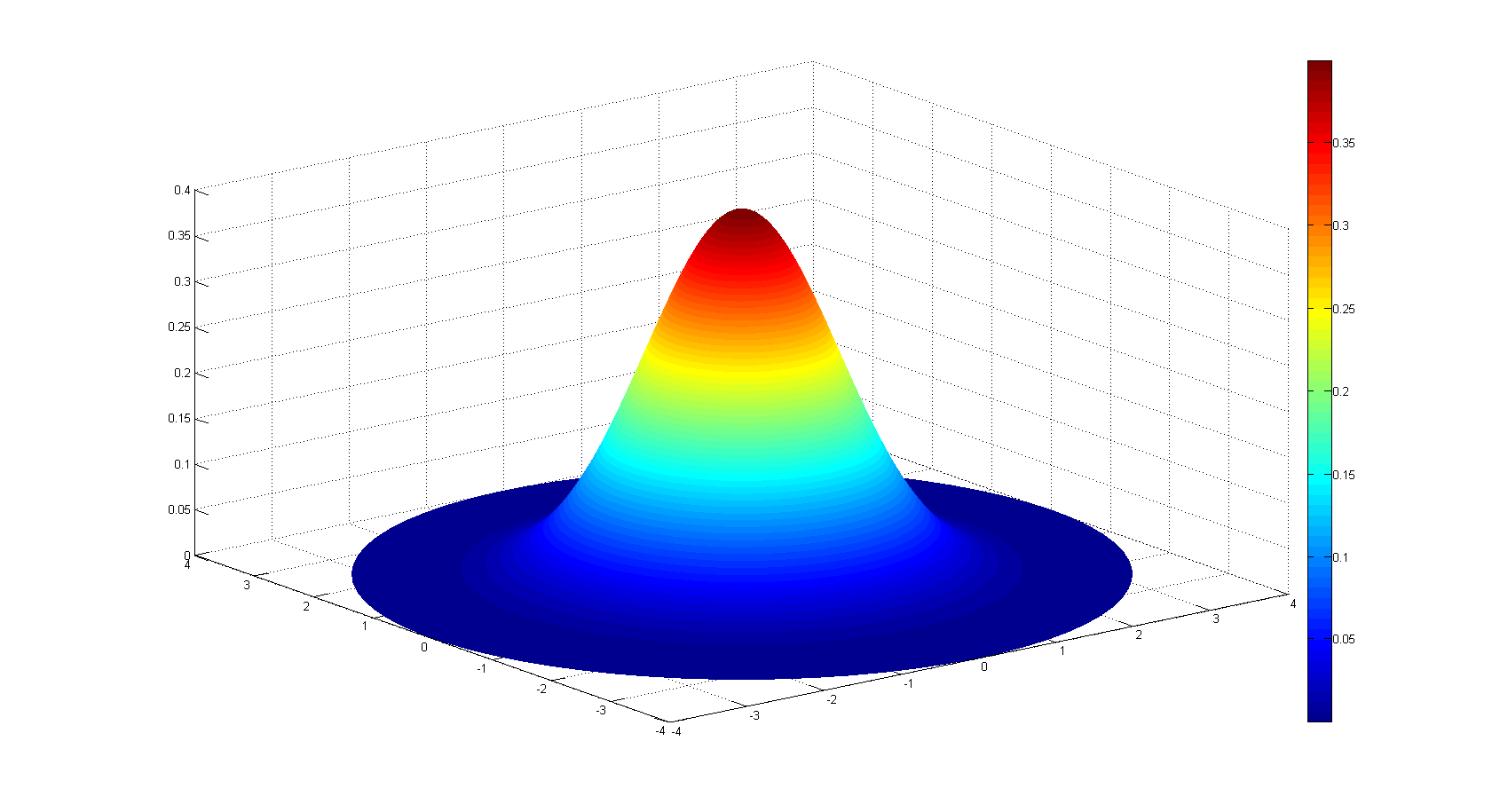


Figure S2. The initial beam profile of femtosecond laser with Gaussian energy distribution.


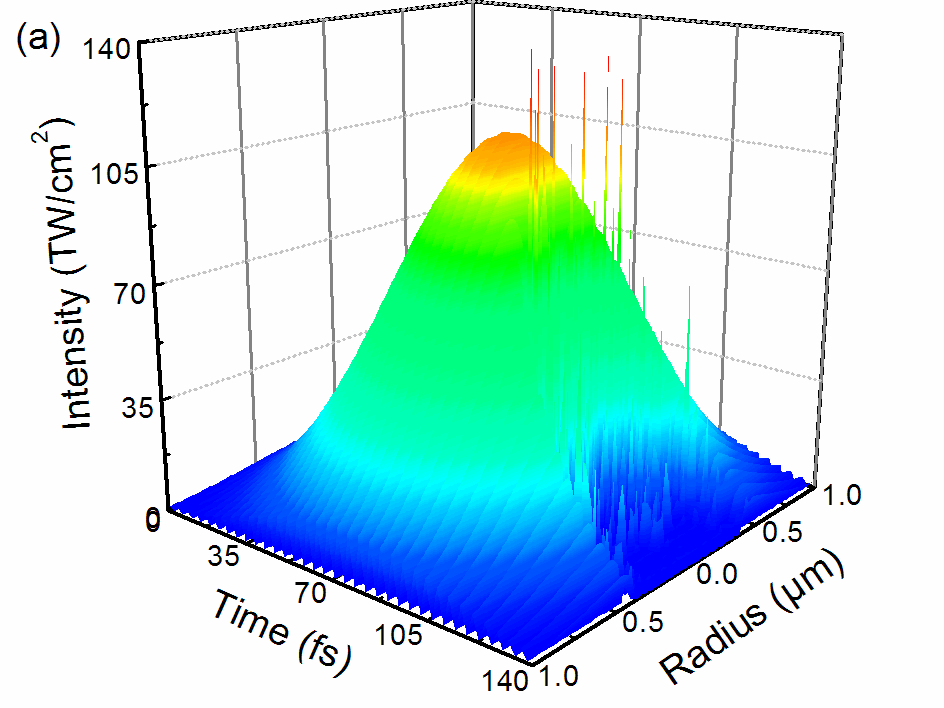


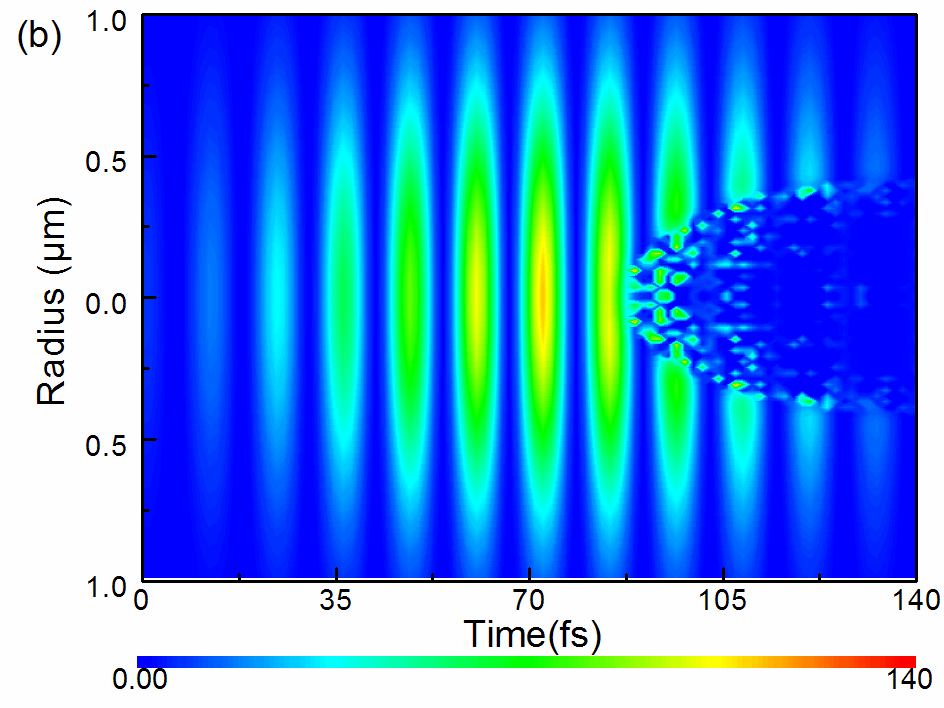


Figure S3. The energy spatial distribution during laser material interactions: (a) 3D beam profile; (b) side view. This case calculates a linearly polarized Gaussian laser pulse (800 nm, 50 fs) ablation of fused silica.

The beam profile, laser ablated profile and chemical profile are exhibited in Figure S4. The initial Gaussian type beam can propagate the sample surface via objective lens. Then, the ablated micro-hole is formed on the surface with irregular spatter around the hole. Finally, the chemical etching solution can remove the ejection to generate perfect microlens.

The initial Gaussian type beam profile is completely symmetric, which is simulated by standard Gaussian equation. However, the actually energy distribution of the femtosecond laser is not perfect Gaussian type beam. When the beam propagates the sample surface via objective lens, the ablated micro-hole is formed on the surface with irregular spatter around the hole, as shown in Figure 2(d). The shape of the micro-hole is not completely circular, and more similar to ellipse. Also, the profile of ablated hole in Figure R1 is not completely symmetric. This phenomenon could be attributed to the main reasons as follows. Firstly, the laser beam is not ideally perpendicular to the surface of sample. It is extremely difficult to ensure the right angle between laser beam and surface of sample [S4]. Secondly, the scanning speed during laser ablation is high (for example 20 mm/s), which may lead to the ablated hole is asymmetric in vertical and horizontal direction with scanning direction. Hence, the ablated hole originated from the symmetric laser beam is not completely symmetric.


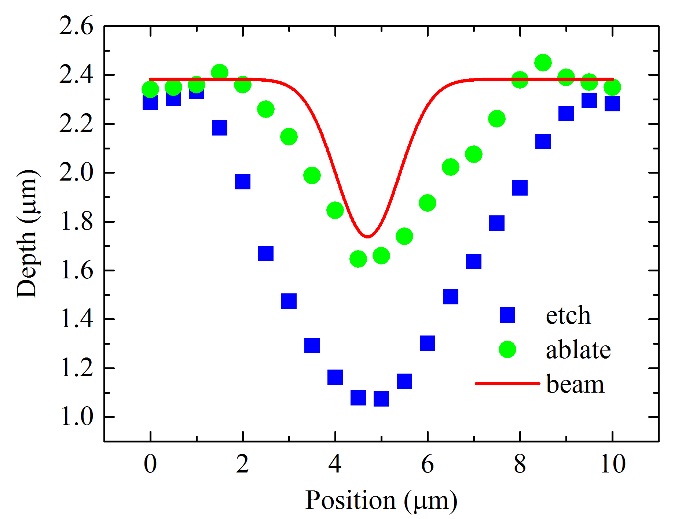


Figure S4. The beam profile, laser ablated profile and chemical etched profile.

After the chemical etching solution treated, the etched profile is different from ablated hole. The chemical etched profile is completely symmetric, also as shown in Figure 2(f). The shape of the hole in SEM image is nearly ideal circular, which could be treated as perfect microlens. The grooves and protrusions around the ablated hole are generated from the nonlinear process. These asymmetric structures and defects will enlarge the interface of the sample’s surface and etching solution, which results in accelerating the etching process in the ablated hole. Finally, the ideally symmetric chemical etched hole is formed, even though it is originated from asymmetric ablated hole.

Generally, after the femtosecond laser ablation of wide band gap materials, craters with a flat-bottom should be formed [S5-S7], which are also confirmed by the plasma model combined with an improved two-temperature equation [S8]. Therefore, there is no particular function could fit the shape of the ablated surface with perfect fitting. Figure S5 has exhibited three types of function to fit the shape of the surface. The root mean square (RMS) deviations between the actual and ideal profile are 0.0168 μm, 0.0167 μm and 0.0301 μm for the Gaussian, Parabolic and Spherical functions, respectively. Compared with the Gaussian and Spherical fitting function, the profile of the fabricated microlens fit better with the parabolic profile. Therefore, the parabolic fitting function with minimum value of RMS is chosen in this study.


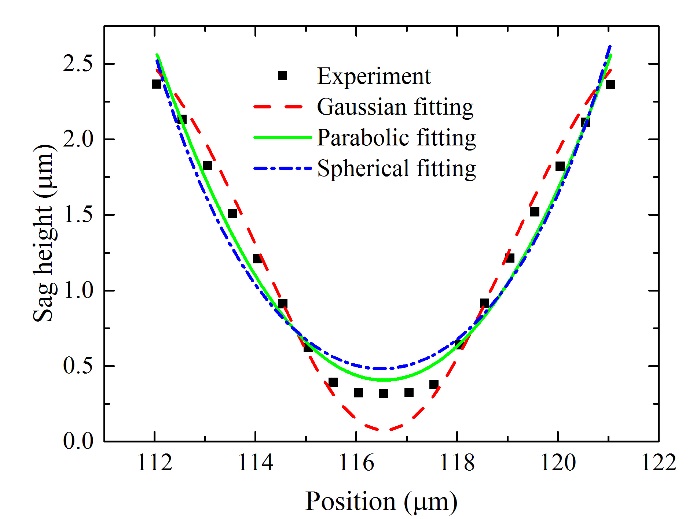


Figure S5.Experimental data for surface profile with the Gaussian function, Parabolic function and Spherical function fitting curve, respectively.

## References

[S1] Zhao, M. J. *et al*. Controllable high-throughput high-quality femtosecond laser-enhanced chemical etching by temporal pulse shaping based on electron density control. *Sci. Rep*. **5**, 13202 (2015).

[S2] Zhang, F. *et al*. Temperature effects on the geometry during the formation of micro-holes fabricated by femtosecond laser in PMMA. *Opt. Laser Technol*. **100**, 256-260 (2018).

[S3] Yuan, Y. P., Jiang, L., Li, X., Wang, C. & Lu, Y. F., Adjustment of ablation shapes and subwavelength ripples based on electron dyncmics control by designing femtosecond laser pulse trains. *J. Appl. Phys*. **112**, 103103 (2012).

[S4] Hu, Y. et al. Cost-efficient and flexible fabrication of rectangular-shaped microlens arrays with controllable aspect ratio and spherical morphology. *Appl. Surf. Sci*. 292, 285-290 (2014)

[S5] Bonse, J., Munz, M. & Sturm, H., Scanning force microscopic investigations of the femtosecond laser pulse irradiation of indium phosphide in air. *IEEE T. Nanotechnol*. **3**, 358-367 (2004).

[S6] Wu, Z. *et al*. Morphological investigation at the front and rear surfaces of fused silica processed with femtosecond laser pulses in air. *Opt. Express* **10**, 1244-1249 (2002).

[S7] Dumitru, G., Romano, V., Weber, H. P., Sentis, M. & Marine, W., Femtosecond ablation of ultrahard materials. *Appl. Phys. A-Mater*. **74**, 729-739 (2002).

[S8] Jiang, L. & Tsai, H. L., A plasma model combined with an improved two-temperature equation for ultrafast laser ablation of dielectrics. *J. Appl. Phys*. **104**, 151104 (2008).
